# A systematic review on the use of healthcare services by undocumented migrants in Europe

**DOI:** 10.1186/s12913-018-2838-y

**Published:** 2018-01-18

**Authors:** Marjolein Winters, Bernd Rechel, Lea de Jong, Milena Pavlova

**Affiliations:** 1 Department of Health Services Research, Maastricht University, PO Box 616, 6200 MD Maastricht, The Netherlands; 2 European Observatory on Health Systems and Policies, London School of Hygiene and Tropical Medicine, Keppel Street, London, WC1E 7HT England; 3 Department of Health Services Research, CAPHRI, Faculty of Health, Medicine and Life Sciences, Maastricht University, PO Box 616, 6200 MD Maastricht, The Netherlands

**Keywords:** Utilization, Healthcare services, Undocumented migrants, Europe, Systematic review

## Abstract

**Background:**

Undocumented migrants face particular challenges in accessing healthcare services in many European countries. The aim of this study was to systematically review the academic literature on the utilization of healthcare services by undocumented migrants in Europe.

**Methods:**

The databases Embase, Medline, Global Health and Cinahl Plus were searched systematically to identify quantitative, qualitative and mixed methods studies published in 2007–2017.

**Results:**

A total of 908 articles were retrieved. Deletion of duplicates left 531. After screening titles, abstracts and full texts according to pre-defined inclusion and exclusion criteria, 29 articles were included in the review. Overall, quantitative studies showed an underutilization of different types of healthcare services by undocumented migrants. Qualitative studies reported that, even when care was received, it was often inadequate or insufficient, and that many undocumented migrants were unfamiliar with their entitlements and faced barriers in utilizing healthcare services.

**Conclusions:**

Although it is difficult to generalize findings from the included studies due to methodological differences, they provide further evidence that undocumented migrants in Europe face particular problems in utilizing healthcare services.

**Electronic supplementary material:**

The online version of this article (10.1186/s12913-018-2838-y) contains supplementary material, which is available to authorized users.

## Background

Current data on the number of undocumented migrants in the European Union (EU) are, by necessity, characterized by inaccuracy and low reliability due to their legal status and incomplete data [1]. Nevertheless, in 2008, a study estimated that 1.9 to 3.8 million undocumented migrants resided in the EU, accounting for 0.39% to 0.77% of the total population and 7% to 13% of the ‘foreign’ population [2].

The term ‘undocumented migrants’ is generally agreed to refer to third-country nationals without a valid permit authorizing them to reside in EU member states. This includes those who have been unsuccessful in asylum procedures (rejected asylum-seekers) or those who have violated the terms of their visas (‘over-stayers’), as well as those who have entered the country illegally [3].

Undocumented migrants have been identified as a particularly vulnerable population facing a number of health risks [4]. National policies on entitlements of undocumented migrants to healthcare services differ widely between EU member states, with many restricting access to different degrees. For example, Cuadra grouped the EU member states in three clusters based on the level of entitlement of undocumented migrants to healthcare services in 2012. Cluster 1 comprised EU member states (e.g. Finland, Ireland) with so-called ‘less than minimum rights’, restricting entitlements to a degree that makes even emergency care inaccessible for undocumented migrants, as they cannot afford paying for it. Cluster 2 included EU member states with ‘minimum rights’ (e.g. Germany, Denmark), where entitlements include access to emergency care, or care specified in terms such as ‘immediate’ or ‘urgent’. Cluster 3 included EU member states with ‘more than minimum rights’ of access to healthcare (e.g. Italy, Portugal), authorizing undocumented migrants to healthcare services beyond emergency care, in particular primary and secondary care [3]. While entitlements in these countries have been shifting, this categorization illustrates that undocumented migrants meet different barriers to access to healthcare services depending on the host country; however, some barriers, such as a fear of deportation, can be assumed to be common.

Analyses of healthcare services utilization by undocumented migrants are often confronted by inconsistent terminology and incomparable or incomplete data. For example, the different EU member states themselves frequently do not collect disaggregated information such as gender and age, which limits the specificity and therefore the comparability of necessary data [1].

Overall, the area is under-researched even though a few review studies can be identified. A scoping review on health and access to care for undocumented migrants in Europe underlined the need for more and better-quality research regarding undocumented migrants [5]. Another review that aimed to identify the health status of undocumented migrants in Europe, reported that undocumented migrants are less likely to receive adequate healthcare services and to access important preventive healthcare services [6].

Although there are reviews focussed on healthcare services access among undocumented migrants, to the best of our knowledge, no systematic review on healthcare services utilization of undocumented migrants has yet been undertaken. This systematic review aimed to fill this gap.

## Methods

The aim of this study was to systematically review the academic literature on the utilization of healthcare services by undocumented migrants in Europe. The literature search was conducted in April 2017 and re-run at the end of May 2017. The review was carried out in accordance with the Preferred Reporting Items for Systematic Reviews and Meta-Analyses (PRISMA) guidelines [7]. A librarian was consulted to improve the search strategy.

As the volume of literature in the topic area is still quite small, we decided to include qualitative, quantitative and mixed-methods studies, provided they were based on original research and published in peer-reviewed journals. All studies included: 1) were published in English, Dutch or German; 2) covered the subject of humans; 3) were published in 2007 or later. Only studies conducted in EU-28 and European Free Trade Association (EFTA) countries were taken into consideration. Studies also had to include at least a partial focus on undocumented migrants, rather than migrants in general. Studies were excluded if they focussed primarily on the screening for specific conditions (e.g. HIV/Aids or Hepatitis B) or on healthcare outcomes. Finally, studies that only focussed on legislation and barriers to access, were also excluded.

Searches were performed in the databases Embase, Medline, Global Health and Cinahl Plus. The initial search was conducted in Embase and consisted of free text terms with truncations (*) and Medical Subject Headings (MeSH), which were also exploded (exp). The search terms were then connected with the Boolean operators AND and OR. Additionally, the two concepts ‘undocumented’ and ‘migrants’ and their synonyms were connected with the adjacent operators with a distance space of 10 (adj10) to cover more search results. The search was then spread out to the Medline and Global Health databases and slightly adjusted for the Cinahl Plus database, although covering the same content with database-specific modifications if necessary. All retrieved references were imported into Endnote, after which duplicates were deleted. The detailed search query for the databases can be found as supplementary material in Additional file 1.

A first selection of articles, based on titles and abstracts, was undertaken by two authors independently. When both agreed that inclusion criteria were met, studies were included in the next step. The second selection was based on full-text screening and checking the reference lists of included studies. The method of qualitative content analysis was applied to extract and summarize all relevant data in Excel. Moreover, the quality of quantitative studies was assessed using the Quality Assessment Tool for Observation and Cross-sectional Studies [8]. This tool encompasses 14 questions, according to which studies were rated as ‘good’, ‘fair’, or ‘poor’. For qualitative studies, the Critical Appraisal Skills Programme was used, [9] consisting of a 10-item questionnaire. We applied the same rating as for the quantitative study assessment tool to ensure comparability. To assess the quality of mixed-methods studies, both tools were used.

The quality of our own systematic review was ensured by making use of the PRISMA 2009 checklist [10] and can be found in Additional file 2.

## Results

**Fig. 1:**
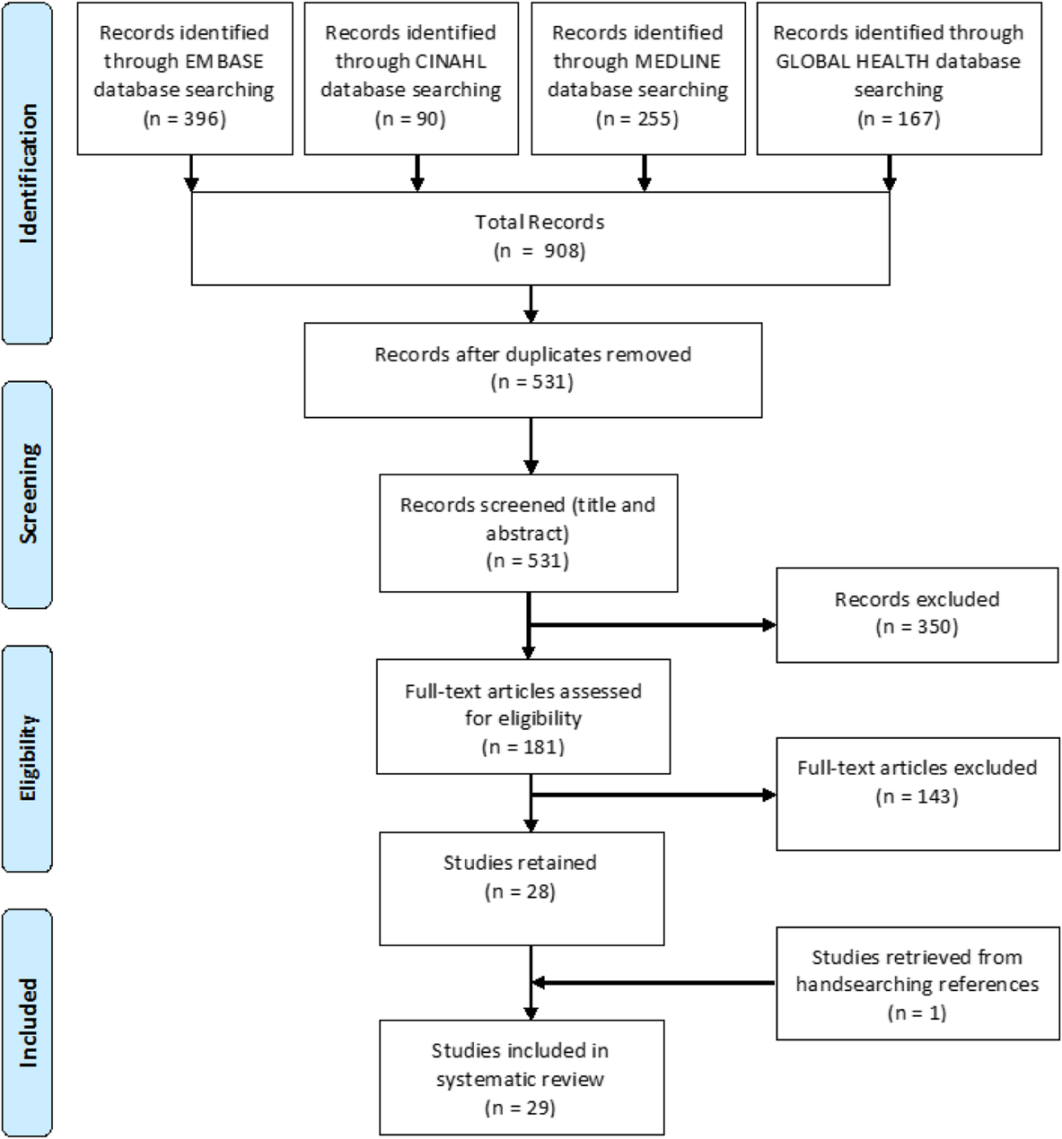
PRISMA Flowchart

A total of 908 articles were retrieved from the literature search, with 531 articles remaining after deletion of duplicates. After title and abstract screening, 181 references remained and were included in full-text screening. After this step, 28 articles were retained and one article was added that was identified through exploring the reference lists of included studies, as illustrated in Fig. 1.

Table 1 shows the characteristics of the included studies. Single-country studies covered 10 European countries, namely Italy (*n* = 6), Denmark (*n* = 2), England (*n* = 2), Switzerland (*n* = 1), Germany (*n* = 4), France (*n* = 1), the Netherlands (*n* = 5), Portugal (*n* = 2), Spain (*n* = 2) and Belgium (*n* = 1), while three studies covered several countries. The types of healthcare services investigated included primary healthcare services (*n* = 19), hospital services (*n* = 19), outpatient specialist services (*n* = 17), accident and emergency (A&E) services (*n* = 15), or were not explicitly defined (*n* = 1). Most of the studies explored utilization of several types of healthcare services.

**Table 1:** Description of included studies

	*N* = 29	
	*n* (%)	References
General characteristics of the study		
Receiving countries		
Italy	6 (21)	[16, 20–22, 30, 32]
Denmark	2 (7)	[13, 36]
England	1 (3)	[15]
Switzerland	2 (7)	[31, 34]
Germany	4 (14)	[21, 23, 24, 37]
France	1 (3)	[35]
The Netherlands	5 (17)	[18, 19, 25, 26, 29]
Portugal	2 (7)	[11, 12]
Spain	2 (7)	[14, 38]
Belgium	1 (3)	[17]
Multi-country analysis^a^	1 (3)	[27]
Multi-country analysis^b^	2 (10)	[28, 39]
Type of health services		
Primary healthcare services	19 (66)	[11–21, 23, 25–27, 29, 36–38]
Hospital services	19 (66)	[11, 12, 14–17, 23, 24, 27, 29–38]
Outpatient specialist services	17 (59)	[12, 14–16, 18, 19, 23, 24, 26–29, 34, 36–39]
A&E services	15 (48)	[11, 12, 14–18, 23, 26, 27, 33, 35–38]
Not defined	1 (3)	[17]
Characteristics of undocumented migrants in the study		
Number of undocumented migrants included		
Up to 100	6 (21)	[14, 15, 17, 36, 38, 39]
Between 100 and 1000	18 (62)	[11–13, 16–19, 23–29, 32–35]
Over 1000	5 (17)	[20, 21, 30, 31, 37]
Percentage of undocumented migrants of total sample size		
0–9	4 (14)	[31–33, 39]
10–49	8 (28)	[11, 12, 17, 19, 24, 28, 35, 38]
50–99	2 (7)	[25, 37]
100	15 (52)	[13–18, 20, 23, 26, 27, 29, 30, 34–36]
Information on place of birth		
Specific country of birth	6 (21)	[14, 16, 17, 21, 35, 36]
Specific region of birth	13 (45)	[12, 13, 20, 23, 24, 26, 29–35]
Country and region of birth	5 (17)	[11, 18, 25, 37, 38]
Non-specific	5 (18)	[15, 17, 27, 28, 39]
Information on type of migration		
Yes	6 (21)	[14, 18, 26, 34–36]
No	23 (79)	[11–13, 15–17, 19–25, 27–33, 37–39]
Methodological characteristics		
Study design		
Quantitative	20 (69)	[11–13, 16–22, 25, 26, 28–35, 37, 38]
Cross-sectional	18 (62)	[11–13, 16, 17, 19–22, 28–33, 35, 37, 38]
Cohort	2 (7)	[25, 34]
Qualitative	7 (24)	[14, 15, 23, 24, 27, 36, 39]
Mixed methods	2 (7)	[18, 26]
Data source		
Registry	9 (31)	[13, 19–22, 25, 30, 32, 37]
Survey	8 (28)	[11, 12, 16, 17, 28, 34, 35, 38]
Registry and survey	3 (10)	[29, 31, 33]
Interview	4 (14)	[14, 15, 27, 39]
Observation	0 (0)	
Interview and observation	3 (10)	[23, 24, 36]
Multiple data sources	2 (7)	[18, 26]
Adjustment		
Defined	13 (45)	[11, 12, 18–20, 22, 25, 29, 31–35]
Not done/not applicable	16 (55)	[13–17, 21, 23, 24, 26–28, 30, 36–39]

^a^Includes Finland, Sweden, Austria, Belgium, Denmark, Germany, Greece, Hungary, Lithuania, Poland, the UK, France, Italy, the Netherlands, Portugal and Spain

^b^Includes Austria, Belgium, Czech Republic, France, Italy, Germany, Hungary, Ireland, The Netherlands, Poland, Portugal, Spain, Sweden and the UK

The number of undocumented migrants included in the studies differed widely. For the qualitative studies, the lowest number of participants was ten and the highest 240. In the quantitative studies, the number of participants ranged from 11 to 10,254. The two mixed methods studies included each 100 undocumented migrants. Of the total sample size, undocumented migrants accounted for less than 9% in four studies, 10–49% in eight studies, 50–99% in two studies and for all participants in 15 studies. Almost half of the studies reported a specific region of birth (*n* = 14), six reported a specific country of birth, four reported on both country and region of birth, and five studies did not report any information on place of birth. Of all included studies, only six reported information on type of migration (e.g. economic or political).

Most studies had a quantitative design (*n* = 20), of which 18 were cross-sectional and two were cohort studies. The data sources of the quantitative studies consisted of registry (*n* = 9), survey (*n* = 8) or a combination of both registry and survey data (*n* = 3). Seven qualitative designs were applied in the included studies, using interviews (*n* = 4) or a combination of interviews and observations (*n* = 3). Only two mixed-methods studies were included, which both used a combination of surveys and interviews. Defined adjustments (e.g. age, gender, socioeconomic status) were found in most of the quantitative studies (*n* = 13), but almost half (*n* = 10) did not perform any statistical analysis for possible confounders or were unable to do so. The number of quantitative, qualitative and mixed-methods articles and the types of healthcare services explored are summarized and presented in Table 2.

**Table 2:** Use of healthcare services of quantitative, qualitative, mixed methods studies

	Quantitative		Qualitative		Mixed methods		Overall
	*N*	References	*N*	References	*N*	References	*N*
Primary healthcare	11	[11–13, 16, 17, 19–21, 25, 29, 37]	6	[14, 15, 24, 27, 36, 39]	2	[18, 26]	19
Hospitalization	12	[11, 12, 16, 17, 29–35, 37]	7	[14, 15, 23, 24, 27, 28, 36, 39]	0		19
Outpatient specialist	8	[11, 12, 16, 19, 22, 28, 29, 37]	7	[14, 23]	2	[18, 26]	17
A&E	7	[11, 12, 16, 17, 34, 35, 37]	6	[14, 15, 24, 27, 28, 36, 39]	2	[18, 26]	15
Not defined	1	[38]	0		0		1

Key findings on the use of healthcare services are reported in Table 3.

**Table 3:** Key findings on undocumented migrants^a^

	Quantitative		Qualitative		Mixed Methods		Overall
	*N*	Reference	*N*	Reference	*N*	Reference	
Underutilization							
Primary healthcare	6	[11–13, 16, 17, 19]	3	[14, 15, 39]	1	[18]	10
Hospitalization							0
Outpatient specialist	1	[25]	1	[27]			2
A&E			1	[36]			1
General	1	[36]					1
Inadequate care							
Maternal	4	[25, 31, 32, 34]	2	[23, 24]	1	[26]	7
Other/general care							0
No difference in utilization	1	[38]					1

^a^This table only represents 20 studies, as the other nine studies do not assess criteria presented in the table

### Primary healthcare services

Overall, several studies found a low utilization rate of primary healthcare services by undocumented migrants in comparison to documented migrants. Both qualitative and quantitative studies, conducted in Italy, Portugal, England and Denmark, reported that undocumented migrants were less likely to seek primary healthcare services than regular migrants [11–15]. Several other studies also reported fewer contacts or consultations with general practitioners by undocumented migrants compared to registered migrants or the native population [16–19].

A few studies provided details on the exact number of consultations at primary healthcare services and trends in consultations by undocumented migrants. First, a study conducted in Milan, the city with the largest foreign-born population in Italy after Rome, reported that 10,571 undocumented migrants sought care at a primary care association in 2000–2001. Unofficial estimates for 2003 ranged between 20,000 and 800,000 undocumented migrants in Italy [20].

Second, the humanitarian consultation hour, provided by the City of Frankfurt am Main, treats people without health insurance or undocumented migrants for free since 2001. This study reported that the amount of consultations doubled between 2008 (*n* = 673) and 2009 (*n* = 1154) and has been rising steadily since. The majority of these patients came from Africa and since 2008, an increase of undocumented patients from Bulgaria and Romania has been recorded [21].

Finally, in Italy, about 51,000 people were estimated to be homeless in 2014, of which 58% were migrants [22]. An outpatient clinic reported that the visits and revisits were greater for homeless undocumented migrants than for homeless registered migrants between 2007 and 2011, highlighting the importance of clinics for marginalized people to migrants who are not entitled to attend an institutional source of care [22].

### Outpatient specialist services

Findings on maternal healthcare in outpatient specialist services were largely consistent across countries. Two qualitative studies in a Berlin clinic reported infrequent prenatal care, as most undocumented women only appeared in the third trimester. They also found that the overall quality and quantity of maternal care is limited compared to documented migrants [23, 24], whilst two Dutch studies reported that, compared to documented women, undocumented women came for consultations later in their pregnancy, received care elsewhere [25], and had high abortion rates [26].

Two studies on mental healthcare, both conducted in several European countries, reported slightly different results. One qualitative study (e.g. the Netherlands) found that being documented may restrict the care available for further mental treatment, such as psychiatric care [27], whilst a quantitative study reported that undocumented migrants had a high prevalence of post-traumatic stress disorder (PTSD) and many received individual or group psychotherapy in several countries [28].

### Hospital services

Most studies on the use of hospital services by undocumented migrants used retrospective cross-sectional data (*n* = 9). Two studies, conducted in the Netherlands and Belgium, found that hospital services were the type of healthcare service most often used by undocumented migrants, followed by general practitioners, due to barriers of registering with general practitioners [17, 29].

Several studies focussed on the use of hospital services in Italy, the country which was placed at the top of the EU list of undocumented migrants in 2008 [16]. One hospital located in Palermo analysed day-hospital admissions of undocumented migrants between 2003 and 2009. The sample population consisted of 1758 undocumented migrants, representing 7.4% of potential users. More than half of these migrants were African and used hospital services most often for gastroenterological diseases, followed by infectious and parasitic diseases [30].

An immigrant outpatient clinic in the Italian region Apulia also reported a majority of African undocumented patients and showed that 61 of the 256 participants from their clinic were once admitted to a hospital (24%) [16].

Studies on the use of maternal healthcare often reported major gaps and challenges. A hospital in Switzerland found a much lower use in medication or contraception among undocumented women compared to documented women [31], while a cross sectional study in Italy found more induced abortions among undocumented women [32]. A French cohort study reported that almost half of undocumented women received inadequate prenatal care (e.g. only half of the recommended visits or no first-trimester examination) [14, 33], while another cohort study in Geneva found that undocumented migrant women had more unintended pregnancies, used preventive measures less frequently and delayed prenatal care more than legal residents [34].

### A&E services

Only a few studies elaborated on the use of A&E services by undocumented migrants, of which two were Italian. In the Apulia region, 23% of undocumented migrants of an outpatient clinic had to be referred to emergency services [16], whereas a hospital in Bologna reported that admission of undocumented migrants with emergency features was very frequent in 1999 (43% of overall admissions), but rapidly declined in the following years [35]. A quantitative study, using patient files of a non-governmental organization (NGO) in Copenhagen, found that 0.5% of all 2088 visits (*n* = 11) were referred acutely or sub-acutely to a hospital to be tested for tuberculosis [13].

Furthermore, one Emergency Room nurse from Denmark reported in a qualitative study the same general conclusion regarding underutilization of primary healthcare services as for A&E services: ‘He was reddish and had a fever, so we recommended that he was hospitalised and treated with intravenous antibiotics. But he didn’t want that. So in the end we patched up his wounds and then sent him away with a prescription for penicillin.’ [36].

### Additional findings

Results on non-specific or general utilization of healthcare services were given by three studies. A quantitative German study conducted in Berlin, Cologne and Bonn found that the patterns of healthcare utilization by undocumented migrants changed in 2006–2007, which was attributed to EU enlargement to the East [37].

In comparison to most of the other included studies, a quantitative Spanish study did not find differences in the utilization of health services by legal status in 2002. However, it did find that having 12 or more years of schooling, having university education, living in Spain for five years or more and having a stable contract compared to a temporal or no work contract, were all associated with higher utilization of healthcare services [38].

Finally, in accordance with many of the other studies, a qualitative Danish study reported that the majority of undocumented migrants participating recounted situations where they had avoided or postponed any contact with healthcare professionals [36].

### Quality assessment.

Of all studies (*n* = 29), 18 were rated as ‘good’, whilst 11 were rated as ‘fair’. No study was rated as ‘poor’. Studies that provided relevant information for all quality questions were rated as ‘good’. Most quantitative studies rated as ‘fair’ were inadequate in reducing or controlling for any bias, such as selection or information bias, whilst qualitative studies rated as ‘fair’ often lacked adequate external validity.

## Discussion

To the best of our knowledge, this study is the first systematic review on the use of healthcare services by undocumented migrants in Europe. Most included studies reported an underutilization of healthcare services in general by undocumented migrants compared to documented migrants, while one quantitative study did not report any differences between these groups [38]. Several quantitative studies focussing on primary healthcare services found fewer contacts or consultations with general practitioners [16–19], whilst qualitative studies pointed out that undocumented migrants rather visit a clinic for free primary care than register with a general practitioner [13, 36, 39]. Two quantitative studies conducted in different countries report that undocumented migrants were more likely to go to a hospital than to a general practitioner [17, 29]. Potential explanations for these findings are the fear of being deported and the limited entitlements to healthcare. For example, in many countries (e.g. Germany, Denmark, Belgium) undocumented migrants are only provided with access to emergency care or sometimes to services for specific conditions (e.g. infectious diseases) or specific needs (e.g. maternal healthcare) [6]. Yet, even with regard to maternal healthcare, to which undocumented migrants are entitled in many European countries, a number of studies found underutilization and inadequate care (e.g. the late appearance of pregnant undocumented women in a clinic) [14, 23, 24, 32]. Furthermore, the differences between the countries regarding healthcare services provision to undocumented migrants could be due to differences in entitlements to healthcare, divergent interpretations of concepts such as ‘basic healthcare’, ‘right to healthcare’ and ‘healthcare accessibility’ or lack of awareness of legal requirements for delivering care to undocumented migrants [27]. In 2017, France, Italy and the Netherlands provided access to healthcare to undocumented migrants on the same basis as to authorised residents, whilst Belgium and Germany provided access to limited healthcare services (e.g. limited in time or in terms of type of health service) and Slovenia provided no access to healthcare services at all, except for urgent medical assistance [40].

Almost all included studies point to a large gap between entitlements and utilization of healthcare services. Qualitative studies with healthcare professionals or undocumented migrants found that many undocumented migrants do not seek healthcare services when needed due to a number of barriers, such as fear, lacking awareness of entitlements, or socioeconomic reasons [5]. This points to missing links between official policies and practice on the ground, something termed ‘implementation gap’ [3]. This gap is due to different factors, such as ambiguities for doctors and patients when the need for treatment may not be acute or inconsistencies between formal access and legislation [41]. Furthermore, as emphasized by the EUropean Refugees-HUman Movement and Advisory Network (EUR-HUMAN), healthcare practitioners may lose their licence or face criminal charges when providing care to undocumented migrants [42]. Reasons for the underutilisation of healthcare services can also be found outside the healthcare system, due to widespread public misperceptions of migrants and their use of social and healthcare services. In the United Kingdom, for example, approximately 75% of survey respondents were in favour of reducing immigration in 2013, mainly due to media campaigns against immigration and the alleged misuse of the British social and healthcare system [43].

None of the countries providing less than minimum rights to healthcare services were found in our search, while most studies included in our review were on countries providing more than minimum rights to healthcare, such as Italy, Portugal and the Netherlands [3]. This could be explained by how well established migrant health research is in the countries or the current basic norms and institutions of the countries. In addition, the number of undocumented migrants who reside in these countries differs widely. For example, it is estimated that there were between 500,000 and 700,000 undocumented migrants in Italy in 2008 [16], while estimates for Denmark ranged from 1000 to 5000 undocumented migrants in 2011 [36].

Overall, undocumented migrants seem to be exceptionally vulnerable, not only because they face numerous barriers in accessing and utilizing healthcare services, but also because their other basic human needs are not met [44, 45]. Data regarding the utilization of healthcare services by undocumented migrants is still scarce due to many reasons, such as ethical considerations or lack of accurate registration regarding healthcare services. Further research is also needed in countries which provide ‘less than minimum rights’ to healthcare to undocumented migrants, as they can become particularly vulnerable in these countries.

### Limitations

This study has several limitations. First, single-country studies from only 10 European countries were included in this review, out of a possible 28 EU and 4 EFTA member states, while there were only three studies performing multi-country analyses. This means that great caution needs to be exercised in making any generalizations from the findings of this review to undocumented migrants in Europe generally.

Second, the methods, scope and quality of the included studies differed widely. Some studies did not focus primarily on undocumented migrants and therefore only had a very small sample size of this group of migrants. Differences were particularly striking between the included quantitative studies, in which the number of participants ranged from 11 to 10,254 undocumented migrants, limiting the generalizability of any findings.

Third, some studies adjusted for possible confounders, whilst others did not mention or correct for any form of bias. In addition, most of the quantitative studies had a cross-sectional design without a comparison group (*n* = 23), whereas only two studies were using cohorts. However, it needs to be acknowledged that undocumented migrants are a population which is very difficult to reach [24]. Most qualitative studies made use of snowball or purposive sampling, with the inherent risk for selection bias.

Finally, we did not search for grey literature, nor did we consider publications in other languages apart from English, Dutch or German or include any studies published before 2007. Broadening the scope of the review would have yielded additional relevant results, such as country-specific data or time trends regarding utilization of healthcare services.

## Conclusion

This article presents the findings of recent quantitative, qualitative and mixed-methods studies concerning the utilization of healthcare services by undocumented migrants in Europe. In general, undocumented migrants seem to use different types of healthcare services less often than legal residents in most of the European countries in which studies were conducted so far. Even when care is utilized, it often seems to be inadequate or insufficient.

However, given the limited number of studies and countries covered, as well as the differences in their methods, scope and quality, conclusions have to be drawn with great caution. Yet, it seems plausible that a gap exists between entitlements to healthcare services (in those countries in which they exist) and the utilization of these services. There is a need for more studies to be conducted on healthcare services utilization by undocumented migrants in Europe, wherever possible with a larger sample size and including control groups. This would provide firmer knowledge on the barriers to healthcare services utilization that will need to be overcome.

## Additional files

Additional file 1: Search strategy for Embase, Medline, Global Health and Cinahl Plus. (PDF 219 kb)

Additional file 2: PRISMA checklist. (DOC 58 kb)

## Abbreviations

A&E: Accident and Emergency
EFTA: European Free Trade Association
EU: European Union
MeSH: Medical Subject Headings
NGO: Non-Governmental Organization
OECD: Organisation for Economic Co-operation and Development
PRISMA: Preferred Reporting Items for Systematic Reviews and Meta-Analyses
PTSD: Post-Traumatic Stress Disorder
