# Supplementary material for: A systematic review on the use of healthcare services by undocumented migrants in Europe
Source: BMC Health Serv Res. 2018 Jan 18;18:30. doi: 10.1186/s12913-018-2838-y (PMC5774156; doi:10.1186/s12913-018-2838-y)
Supplement: Supplementary file 1 — Search strategy for Embase, Medline, Global Health and Cinahl Plus. (PDF 219 kb) [file 12913_2018_2838_MOESM1_ESM.pdf]

Additional file 1

**Embase, Medline and Global Health**

[(undocumented adj10 migr* or undocumented adj10 immigr* or illegal adj10 migr* or illegal adj10 immigr* or irregular adj10 migr* or irregular adj10 immgr* or unauthori#ed adj10 migr* or unauthori#ed adj10 immgr* or clandestine adj10 migr* or clandestine adj10 immigr* or undocumented patient*) OR exp "Undocumented immigrants"/ (MeSH) OR (exp "Transient and migrants"/ (MeSH) AND undocumented or illegal or irregular or unauthori#ed or clandestine) OR (exp "Emigrants and immigrants"/ (MeSH) AND undocumented or illegal or irregular or unauthori#ed or clandestine) OR (exp "Emigration and immigration"/ (MeSH) AND undocumented or illegal or irregular or unauthori#ed or clandestine) OR (undocumented or illegal or irregular or unauthori#ed or clandestine AND migr* or immigr*)] AND [exp "Europe"/ (MeSH) OR exp "European Union"/ (MeSH) OR exp "Europe, Eastern"/ (MeSH) OR (Europe or European Union or EU or European Free Trade Association or EFTA or Euro*) OR (Austria or Belgium or Bulgaria or Croatia or Cyprus or Czech Republic or Denmark or Estonia or Finland or France or Germany or Greece or Hungary or Ireland or Italy or Latvia or Liechtenstein or Lithuania or Luxembourg or Malta or Netherlands or Norway or Poland or Portugal or Romania or Slovakia or Slovenia or Spain or Sweden or Switzerland or United Kingdom or UK or England or Scotland or Wales or Northern Ireland)]

**Cinahl Plus**

[(undocumented N10 migr* or undocumented N10 immigr* or illegal N10 migr* or illegal N10 immigr* or irregular N10 migr* or irregular N10 immgr* or unauthori?ed N10 migr* or unauthori?ed N10 immgr* or clandestine N10 migr* or clandestine N10 immigr* or undocumented patient*) OR exp "Undocumented immigrants"/ (MeSH) OR (exp "Transient and migrants"/ (MeSH) AND undocumented or illegal or irregular or unauthori?ed or clandestine) OR (exp "Emigrants and immigrants"/ (MeSH) AND undocumented or illegal or irregular or unauthori?ed or clandestine) OR (exp "Emigration and immigration"/ (MeSH) AND undocumented or illegal or irregular or unauthori?ed or clandestine) OR (undocumented or illegal or irregular or unauthori?ed or clandestine AND migr* or immigr*)] AND [exp "Europe"/ (MeSH) OR exp "European Union"/ (MeSH) OR exp "Europe, Eastern"/ (MeSH) OR (Europe or European Union or EU or European Free Trade Association or EFTA or Euro*) OR (Austria or Belgium or Bulgaria or Croatia or Cyprus or Czech Republic or Denmark or Estonia or Finland or France or Germany or Greece or Hungary or Ireland or Italy or Latvia or Liechtenstein or Lithuania or Luxembourg or Malta or Netherlands or Norway or Poland or Portugal or Romania or Slovakia or Slovenia or Spain or Sweden or Switzerland or United Kingdom or UK or England or Scotland or Wales or Northern Ireland)]
